# Ligature of the Spermatic Artery for Varicocele

**Published:** 1829-07-01

**Authors:** 


XLVII.

Ligature of the Spermatic Artery for Varicocele.

Various modes of treating varicocele have at various times been had recourse to, but none of them have acquired or deserved any permanent reputation. Blistering, ligature of the spermatic vein, and so forth, are now but seldom employed, and in general, support of the parts and such palliative measures are all that are adopted in the treatment of this inconvenience rather than disease. M. Amussat, of Paris, has recently practised what, as far as we know, is a novel operation for varicocele, namely, the ligature of the spermatic vessels.

The patient, M. Nicolas Rigueri, a Greek, ætatis 30, presented himself to M. Amussat in the month of October last, with a varicocele on the left side as large as the head of a child. Its size was productive of much inconvenience, and obliged him to support it in a linen bag when he wished to walk. The disease had begun at the age of fifteen, without any appreciable cause, and had not been relieved by keeping quiet and soothing local applications. M. Amussat, on examining the case conceived that the only way of curing the disease was by tying the spermatic arteries, and producing the wasting of the testicle. Accordingly, having laid the cord bare for upwards of an inch in length, he dissected for the spermatic arteries, exposed them, although with considerable difficulty, and successively tied both the spermatic trunk, and ten other smaller branches that pass to the testis along the cord. The operation was "extremely delicate and painful," and after its performance the patient had symptoms of reaction which required a copious bleeding, and antiphlogistic measures. On the next day the scrotum was greatly swollen and œdematous, and on the fifth, an abscess, which had formed on the left side of the scrotum, was opened, and a quantity of fetid pus let out. Sloughs of cellular membrane came away, and a large sore occupied the anterior part of the scrotum, which was slow in healing, and remained fistulous for a length of time. The wound made in the operation itself quickly cicatrized. At the time of the report (Feb. 24th) the testicle appeared to be wasted, a large irregular cicatrix, the consequence of the sloughing, occupied the anterior part of the scrotum, and the spermatic veins, so large before, were converted into hard and knotted cords.

The details of this operation will convince any one that it would be quite unjustifiable, unless the varicose condition of the veins was very extensive and severe. But even in such a case we doubt whether castration at once would not be better. The ligature of the spermatic artery cures by producing wasting of the testicle, then why not remove that at once, and effectually remove the disease along with it? We would not wish to be misunderstood; we believe that there are very few cases indeed where either operation is proper, but we also believe that, if either is to be done, castration is preferable to tying the spermatic vessels. The latter, according to M. Amussat himself, is an "extremely delicate and painful operation," is attended with quite as much risk as the former, and after all, is not *sure* to effect the cure of the varicose veins. M. Amussat perceiving the risks which he had run, recommends only tying the main spermatic trunk in future. This might certainly render the proceeding more simple, but would it not render it much less effectual? Surely the anastomoses of the other arteries would still be sufficient to feed the veins.

---

